# Thrombotic thrombocytopenic purpura-hemolytic uremic syndrome (TTP-HUS): a 24-year clinical experience with 178 patients

**DOI:** 10.1186/1756-8722-1-23

**Published:** 2008-12-01

**Authors:** Mark Levandovsky, Danielle Harvey, Primo Lara, Ted Wun

**Affiliations:** 1 Division of Hematology-Oncology, Weill School of Medicine, Cornell University, New York, NY, USA; 2 Department of Public Health Sciences, University of California, Davis School of Medicine, Sacramento, CA, USA; 3 Division of Hematology and Oncology, UC Davis School of Medicine, Sacramento, CA, USA; 4 VA Northern California Health Care System, Sacramento, CA, USA

## Abstract

**Background:**

Thrombotic thrombocytopenic purpura and the hemolytic uremic syndrome (TTP-HUS) are related and uncommon disorders with a high fatality and complication rate if untreated. Plasma exchange therapy has been shown to produce high response rates and improve survival in patients with many forms of TTP-HUS. We performed a retrospective cohort study of 178 consecutively treated patients with TTP-HUS and analyzed whether clinical or laboratory characteristics could predict for important short- and long-term outcome measures.

**Results:**

Overall 30-day mortality was 16% (n = 27). 171 patients (96%) received plasma exchange as the principal treatment, with a mean of 8 exchanges and a mean cumulative infused volume of 42 ± 71 L of fresh frozen plasma. The rate of complete response was 65% or 55% depending on whether this was defined by a platelet count of 100,000/μl or 150,000/μl, respectively. The rate of relapse was 18%. The Clinical Severity Score did not predict for 30-day mortality or relapse. The time to complete response did not predict for relapse. Renal insufficiency at presentation was associated with a decreased risk of relapse, with each unit increase in serum creatinine associated with a 40% decreased odds of relapse. 72% of our cohort had an idiopathic TTP-sporadic HUS, while 17% had an underlying cancer, received a solid organ transplant or were treated with a mitomycin-based therapy. The estimated overall 5-year survival was 55% and was significantly better in those without serious underlying conditions.

**Conclusion:**

Plasma exchange therapy produced both high response and survival rates in this large cohort of patients with TTP-HUS. The Clinical Severity Score did not predict for 30-day mortality or relapse, contrary to our previous findings. Interestingly, the presence of renal insufficiency was associated with a decreased risk of relapse. The most important predictor of mortality was the presence or absence of a serious underlying disorder.

## Background

Thrombotic thrombocytopenic purpura and the hemolytic uremic syndrome are rare, closely-related disorders characterized by microangiopathic hemolytic anemia (MAHA) and thrombocytopenia. Thrombotic thrombocytopenic purpura was first reported by Moschowitz in 1925 and is classically described as a pentad of hemolytic anemia, thrombocytopenia, neurological symptoms, renal involvement, and fever [1-3], although only a minority of patients present with the complete pentad[4,5]. Hemolytic uremic syndrome, first described by Gasser et al. in 1955[6], is often preceded by a diarrheal illness and presents with MAHA and thrombocytopenia and a clinical picture dominated by renal insufficiency. Significant insights into the pathophysiology of these disorders have recently been described. In the early 1980s, ultra large multimers of von Willebrand factor (ULVWF) were found in the plasma of thrombotic thrombocytopenic purpura patients[7]. The presence of ULVWF was ultimately found to be due to a lack of von Willebrand cleaving protease activity, due either to congenital deficiency or an IgG inhibitor [8-10]. This protease has been identified[11,12] and designated ADAMTS13 (A disintegrin and metalloproteinase family with thrombospondin-like motifs) and processes the ULVWF by proteolytic cleavage. Though there are conflicting data, TTP is most often associated with severe deficiencies of ADAMTS13 activity, whereas in HUS activity of this protease is relatively preserved. Further, pathologic differences have been observed between malignancy and chemotherapy associated TTP-HUS as compared to idiopathic/HIV-linked TTP and sporadic HUS [13,14]; the former characterized by both micro and macrovascular fibrin thrombi, as opposed to the microvascular, platelet-rich angiopathy seen in the latter.

Without treatment, thrombotic thrombocytopenic purpura is often a fatal disease, with a mortality rate in excess of 95%[15]. Plasma exchange (PE) has been shown in several case series to produce response rates of approximately 80% and survival rates greater than 90% [16-20]. The effectiveness of plasma exchange was confirmed in a prospective randomized clinical trial by the Canadian Apheresis Study Group, which demonstrated that PE was more effective than simple plasma infusion in the treatment of thrombotic thrombocytopenic purpura[19]. While the role of plasma exchange in malignancy and chemotherapy associated TTP is limited[1,14] the utility of PE in patients with HUS is controversial, given common clinical features and high morbidity of untreated TTP-HUS, withholding PE may be inappropriate[5,18,21]. Present practice at our institution and others[1] is to treat TTP and HUS in a similar fashion.

We report a 24-year experience for 178 patients, predominantly with idiopathic thrombotic thrombocytopenic purpura-hemolytic uremic syndrome (TTP-HUS), treated principally with plasma exchange. This represents one of the largest cohorts of TTP-HUS cases reported in the literature and provides insights into the clinical characteristics at presentation, predictors of response and relapse, and determinants of relapse and mortality.

## Results

There were 178 patients included in this study, of whom 144 (81%) had TTP; the remainder had a diagnosis of HUS. Table 1 presents characteristics of the cohort at the time of diagnosis. About two-thirds of patients were female, with an age range of 1.5 to 85 years. There were fourteen patients 18 years or younger, and 7 patients 10 years or younger. Six patients 18 or younger had a diagnosis of HUS. These patients did not routinely undergo therapeutic plasma exchange at our institution unless they were refractory to supportive measures and at the discretion of the treating pediatric hematologist. Of the 34 patients that carried the primary diagnosis of HUS, 6 were 18 years or younger.

**Table 1:** Patient Characteristics at the Time of Diagnosis

	Number (Percent) or Mean ± SD
Age (years)	49 ± 20
Female	120 (68)
Ethnicity (n = 169)	
White	114 (68)
Hispanic	21 (12)
African American	23 (14)
Asian	7 (4)
Other	4 (2)
Neurological symptoms	99 (59)
Fever	73 (44)
Hemoglobin (g/dl)	9.0 ± 2.1
Platelet count (per μL)	49,000 ± 57,000
Serum lactate dehydrogenase (times upper limit of normal)	6.3 ± 8.1
Serum creatinine (mg/dL)	3.2 ± 2.6
Comorbid conditions	
Cancer	14 (8)
Solid organ transplantation	10 (6)
HIV infection	9 (5)
Mitomycin-based therapy	6 (3)
*E. coli *O157:H7 infection	4 (2)
Sepsis	4 (2)
Hepatitis C	3 (2)

Their ethnic distribution reflected the demographic characteristics of our region, with about 32% non-Caucasian. Twenty-eight percent of the patients had an underlying serious medical disorder, as previously defined. The median platelet count was 31,000/μL, with a range from 2,000 to 383,000/μL; the median hemoglobin level was 9.2 g/dL with a range of 1.7 to 16.0 g/dL. Initial platelet and hemoglobin values were not available on some patients who had been transferred to our institution, many of whom had already begun therapy. Thus, baseline values at our institution did not reflect the values at presentation. The serum creatinine level was less than 1.5 mg/dL in 33% of patients, between 1.5 and 2.5 mg/dL in 25%, and greater than 2.5 mg/dL in 42%. The serum lactate dehydrogenase level (LDH) was elevated above the upper limit of normal in all but 10 (6%) of the 158 patients in whom an LDH level was available at the time of diagnosis. A Clinical Severity Score could be assigned to 168 patients. The mean score was 4.4 ± 1.4, with a median of 4 and a range of 1 to 8.

Plasma exchange was the principal treatment in 171 (97%) patients with the remaining 7 patients receiving either fresh frozen plasma infusion only (n = 2) or only Staphylococcal protein A absorption column therapy (n = 2); no treatment information was available for the remaining 3. Of those patients receiving PE, 19 received fewer than 4 plasma exchanges and charts were not available for 3. Thus, 152 patients were assessable for response to PE. The median number of PE was 8 (range 1–262). The mean cumulative volume of fresh frozen plasma infused was 42 ± 71 L, with a median of 22 L. Corticosteroids were administered in 27% of the patients, anti-platelet agents (aspirin or dipyridamole) in 14%, vincristine in 12%, and dialysis in 20%. Protein absorption column therapy, intravenous immunoglobulins, splenectomy, and fresh frozen plasma infusion without exchange were each used in less than 5% of patients. One patient had been treated with ticlopidine.

Using a cut-off value for the platelet count of 100,000/μL to define a complete response, 124 of 152 (82%) patients responded, including 97 (64%) complete responders and 27 (18%) partial responders (50,000–100,000/μL). There were 28 (18%) treatment failures. Using a platelet count of 150,000/μL to define a complete response, there were 81 (53%) complete responders, 39 (26%) partial responders, and 32 (21%) treatment failures.

Twenty-seven (16%) of the 167 patients for whom we have death information died within the first thirty days following diagnosis, including 1 patient who died of an HIV-related infection, 1 who died of sepsis, and 1 who died of intracranial hemorrhage. Of the 23 patients who died of TTP or HUS, 8 (35%) did not receive at least 3 plasma exchanges (including one who refused treatment) and 1 had an initial complete response. There were 26 deaths after day 30 for which there was information as to cause. Of these, three were attributed to TTP at day 34, 10 months, and 152 months. One late death, at 132 months, was possibly related to TTP. Most of the other late deaths were attributed to cancer, lung, and cardiovascular disease. Of the 125 patients with adequate follow-up data, 23 (18%) relapsed following an initial response.

Table 2 presents the results of the univariate logistic regressions for 30-day mortality and relapse. None of the patient characteristics were significantly associated with 30-day mortality. Interestingly, an increased initial serum creatinine was associated with a lower odds ratio for relapse. Table 3 presents the results of the multivariate models. Serum creatinine was no longer significantly associated with relapse in the multivariate model. Secondary analyses also investigated the univariate association between the number of exchanges needed to achieve a response for both platelet cutoffs (100,000/μL and 150,000/μL) and relapse, restricted to those individuals who had a complete response at the particular cutoff. There was no association for the 100,000/μL cutoff (OR: 1.07; 95% CI: 0.98–1.18; p-value: 0.15). There was, however, an association for the 150,000/μL cutoff, with higher numbers of exchanges needed to reach a complete response associated with a higher risk of relapse (OR: 1.08; 95% CI: 1.003–1.16; p-value: 0.04).

**Table 2:** Univariate associations between patient characteristics and 30-day mortality and relapse*

Variable (unit)	30-Day Mortality OR (95% CI)	p-value	Relapse OR (95% CI)	p-value
Clinical Severity Score	1.29 (0.94–1.79)	0.12	0.87 (0.58–1.31)	0.51
Fever at presentation	0.77 (0.33–1.85)	0.56	0.55 (0.19–1.57)	0.26
Age	1.014 (0.99–1.04)	0.2	0.99 (0.96–1.01)	0.3
Platelet count (per μL)	1.0 (0.99–1.01)	0.94	1.0 (0.98–1.01)	0.72
Hemoglobin (g/dL)	1.03 (0.85–1.25)	0.77	0.93 (0.75–1.16)	0.52
Serum creatinine (mg/dL)	1.10 (0.95–1.28)	0.21	0.61 (0.39–0.94)	0.03

* There were no associations between gender, ethnicity, neurologic symptoms or serum lactate dehydrogenase levels and either 30-day mortality or relapse.

**Table 3:** Multivariate logistic regression models between patient characteristics and 30-day mortality and relapse

	30-Day Mortality			Relapse		
Variable (unit)	OR	95% CI	p-value	OR	95% CI	p-value
Clinical severity score (1 unit)	1.46	0.84–2.54	0.18	1.32	0.64–2.7	0.46
Fever at presentation	0.69	0.24–2.00	0.50	0.28	0.07–1.15	0.08
Age (1 year)	1.007	0.98–1.04	0.64	0.97	0.93–1.01	0.09
Platelets (1/μL)	1.00	0.98–1.02	0.9	1.02	0.99–1.05	0.20
Hemoglobin (1 g/dL)	1.19	0.90–1.58	0.2	1.07	0.81–1.41	0.7
Serum creatinine (1 mg/dL)	1.00	0.80–1.25	0.98	0.53	0.27–1.05	0.07
Lactate dehydrogenase (times upper limit of normal)	1.00	0.95–1.06	0.91	1.02	0.90–1.16	0.72
Neurological symptoms at presentation	1.54	0.41–5.75	0.5	1.25	0.27–5.74	0.77
Female	1.93	0.64–5,.89	0.25	0.92	0.26–3.27	0.9

**Figure 1:**
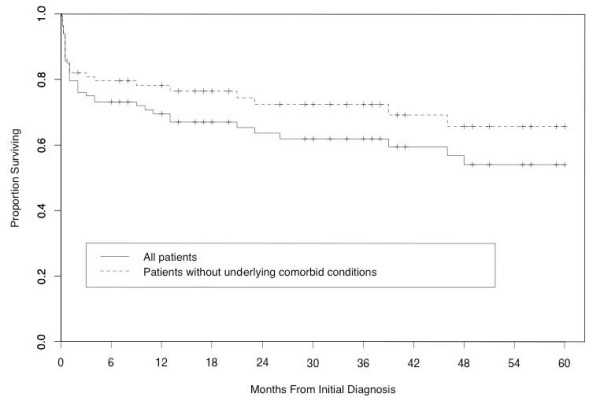
**Kaplan-Meier curve for 5 years of follow-up**. Using either univariate association or multivariate logistic regression model there was no association between CSS and either 30 day mortality or relapse (OR 1.29, p = 0.12; OR 0.87, p = 0.51; OR 1.46, p = 0.18, OR 1.32, p = 0.46, respectivelly).

Figure 1 presents the Kaplan-Meier curves for all individuals included in the death analyses as well as for those individuals (n = 134) without serious underlying disorders, including cancer, organ transplant, HIV/AIDS, or sepsis. The estimated survival probability at 5 years is 54% for all subjects and 66% for those without serious underlying conditions. The estimated survival probability at 10 years is 48% for all subjects and 57% for those without co-morbidities. There was a significant difference in the survival patterns between those with co-morbidities and those without co-morbidities (p < 0.001).

## Discussion and conclusion

This retrospective study represents one of the largest single center clinical experiences with TTP-HUS treated predominantly with plasma exchange. This report affirms findings of other investigators in smaller cohorts of patients. However, this larger analysis contradicts our previous findings that Clinical Severity Score[22] was predictive of mortality and suggests that a longer time to response is associated with a higher risk of relapse. It also suggests that the most important determinant of long-term survival is the presence or absence of a serious underlying medical condition.

The baseline characteristic of our patients is similar to those reported by others[5,20](Table 1). There was a female predominance (68%) and a median age of 49. The ethnicity was reflective of the population of California. After MAHA and thrombocytopenia, neurological symptoms were the next most common manifestation, consistent with other series[5] and further illustrative that the pentad is found only in a minority of the patients. The mean presenting platelet count was higher than in most series[5,20], perhaps reflecting an increased sensitivity to the diagnosis in our region. In contrast to other recent series[20], this present cohort had a lower proportion of patients with serious underlying conditions, drug-related TTP-HUS, pregnancy, or alternative diagnoses. This must be taken into account when interpreting our response and mortality rates.

The overall response rate of 80–83% is similar to the 77% previously reported by our group in a smaller cohort of patients[18], and others in the literature[5,16,19,20,23]. The observed 30-day mortality of 16% is higher than the mortality rate of 10% previously reported by our group[18], but compares favorably to the 21.3% mortality reported by Shamseddine et al[24] In the plasma exchange arm of the Canadian Apheresis Study Group trial, the mortality rate for the plasma exchange group at four weeks was approximately 20%[19]. The majority of our patient cohort (128/178 or 72%) had idiopathic TTP. In this sub-group, the mortality and response rates are similar to the corresponding idiopathic TTP sub-group reported by Vesely et. al. (20% and 83%, respectively)[20].

Our previous analysis[18] had shown that the Clinical Severity Score (CSS) was a potentially useful predictive variable for 30-day mortality. However, the CSS for this expanded patient cohort failed to demonstrate a correlation with either mortality or relapse. These findings are in agreement with those of Vesely et. al. who reported that no clinical or laboratory variable at presentation was predictive or relapse or survival[20].

Although this study did not have adequate power to analyze an association between time to response and death (due to the low number of deaths at 30 days), we were able to examine the association between time to response and relapse. Our data show that a higher number of PE to reach a platelet count of 150,000/μL was associated with a higher risk of relapse; however, the OR was 1.08 and this barely reached statistical significance (p = 0.04). This may reflect the time required to correct ADAMTS13 activity; unfortunately, such data are not available for this cohort. Contemporary studies should examine whether there is any correlation between the time to normalization of ADAMTS13 activity and risk of relapse. If confirmed by others, this would suggest that patients requiring a higher number of PE to reach a complete response might require longer therapy to prevent relapse. However, this hypothesis would need to be tested in a clinical trial. The inverse correlation between serum creatinine and risk of relapse may also reflect ADAMTS13 activity levels.

The overall 5-year survival of about 55% in this cohort was lower than seen in our previous report and long-term data by others[25]. Potential reasons for this include a somewhat older median age than reported by some groups and excess deaths from serious underlying comorbid conditions. However, our data also confirm the better survival in patients treated with plasma exchange without underlying comorbidities, as reported by others[20,26].

There are several important limitations that must be considered when interpreting the results of this study. This was a retrospective study with all of the attendant limitations of such a method. This analysis spans twenty-four years and may be influenced by 1) contextual therapies considered appropriate at various points in time, 2) variability in the health care professionals providing primary treatment and the care in the different hospitals where treatment was delivered, and 3) the different apheresis machines used during the study period. The influence of ancillary treatments on our study parameters was not analyzed because of the small numbers of patients receiving these treatments and the non-standardized means of determining when, why, and how these treatments were administered. Follow-up and mortality data were unavailable for a sizable number of patients because of the number and geographic separation of the serviced hospitals, the high migration (relocation) rates in our region, and the unavailability of some charts. Some of the follow-up data were culled from physician and/or patient interview and thus subject to recall bias. Despite this relatively large cohort of patients, the sample size is small limiting the power to detect small differences among subgroups. Ideally, ADAMTS13 antigen and activity levels would have also provided greater insight into this cohort of patients. Unfortunately, saving plasma from our patients was not routine, and the assays were not available. However, there is yet mixed data regarding the diagnostic and predictive utility of ADAMTS13 activity levels in patients presenting with clinical TTP/HUS[8,10,21,26-29].

During the time period of this study TTP-HUS was, and remains today, a clinical diagnosis[1]. Thus, the results of this study are still pertinent to the care of such patients today. A major determinant of long-term survival is the presence of a serious underlying co-morbidity. These findings also reaffirm that plasma exchange is associated with a high response rate and that no clinical parameters at diagnosis predict for response or survival. Therefore, aggressive plasma exchange should be attempted in all patients with otherwise unexplained MAHA and thrombocytopenia.

## Methods

### Study Population

The study population included consecutive patients with a diagnosis of TTP-HUS referred to the therapeutic apheresis service of the Sacramento Medical Foundation Blood Center (SMFBC) and the University of California Davis Medical Center (UCDMC) from 1978 through 2002. SMFBC (now called Bloodsource) is a not-for-profit community blood center that serves 41 hospitals in a 17-county area of Northern California with an estimated 2.8 million catchment area. From 1984–1988, the UCDMC had an independent therapeutic apheresis service. Prior to 1984, and after 1988, all patients requiring plasma exchange were treated by the SMFBC. The vast majority of patients referred for treatment were seen in the greater Sacramento area at 13 participating hospitals which include large and smaller community hospitals. During this period of time, there were no other apheresis services in the region besides SMFBC and UCDMC. Therefore, all patients referred for apheresis for TTP/HUS should have been treated by one of these two services. All patients in this analysis were required to have microangiopathic hemolytic anemia characterized by schistocytes on the peripheral blood smear and thrombocytopenia (defined as a platelet count < 150,000/μL), with no other identifiable cause for the anemia and thrombocytopenia (e.g., disseminated intravascular coagulation, hypertensive crisis, or eclampsia). All patients had normal prothrombin and activated partial thromboplastin times.

Prior to September 1993, plasma exchanges were performed using the Fenwal CS-3000 Blood Cell Separator or the Haemonetics model V50 machines. After September 1993, all exchanges were performed using the Cobe Spectra.

### Study design and outcome variables

We performed a retrospective cohort study. The primary study outcome was death from TTP-HUS within 30 days of diagnosis and initiation of therapy. The secondary outcomes were overall survival in patients who were followed after hospital discharge, relapse rates and response rates to plasma exchange. We evaluated possible factors that might predict 30-day mortality and relapse. Further, we describe the clinical characteristics of, and ancillary treatments received by, the patients included in this analysis.

### Data collection

A retrospective therapeutic apheresis chart review was conducted using standardized forms and an explicit abstraction process. Data unavailable from the therapeutic apheresis charts were obtained from the actual hospital/medical records, if possible. Quality assurance was performed by reviewing a random sample of medical records. Long term follow-up information after hospital discharge was collected either through review of the appropriate office charts, follow-up questionnaires sent to the referring physician, physician or patient interview, or a combination of the above. Determination of cause of death was done through review of medical records and/or death certificates.

We collected information on demographic characteristics (age, gender, race, date of initial treatment, site of treatment), initial clinical presentation (presence of fever or neurologic abnormalities), laboratory values at the time of initial presentation (hemoglobin, hematocrit, platelet count, serum lactate dehydrogenase, and serum creatinine), number of plasma exchanges required to achieve a complete response (see below), total number of plasma exchange treatments, total volume of plasma infused, concurrent conditions deemed significant (underlying malignancy, solid organ transplants, mitomycin therapy, post-partum state, HIV/AIDS, and *E coli *H:0157 infection), and concurrent ancillary treatments for TTP-HUS (corticosteroids, anti-platelet agents, vincristine, Staphylcoccal protein A adsorption column, intravenous gamma globulins, hemodialysis, peritoneal dialysis, and splenectomy).

As a measure of disease severity, patients were assigned a previously described Clinical Severity Score [22] based on four clinical and laboratory parameters, if available, at the time of presentation (Table 4). The Severity Score incorporates the neurological, renal, and hematological abnormalities and is the sum of all the parameters, with a range of 0–8 points. We had previously found a correlation between this score and 30-day mortality[18].

**Table 4:** Clinical Severity Scoring of Patients with Thrombotic Thrombocytopenic Purpura/Hemolytic Uremic Syndrome[22]

Score	Neurologic Symptoms	Renal Insufficiency	Platelet Count (/μL)	Hemoglobin Level (g/dL)
0	None	None	> 100,000	> 12
1	Confusion, lethargy	Cr* = 1.5–2.5 Proteinuria Hematuria	20,000 – 100,000	9 – 12
2	Seizure, coma, focal deficits	Cr > 2.5 Dialysis	< 20,000	< 9

Abbreviation: Cr: serum creatinine (in mg/dL).

### Treatment

Plasma exchange therapy in our patients was initiated within twenty four hours of diagnosis at 1.5 times the predicted plasma volume for the initial procedure(s). All patients undergoing plasma exchange were treated in a relatively uniform fashion, with daily exchanges until stabilization of the platelet count above 100,000/μL associated with no new or progressive neurological deficits and a declining serum lactate dehydrogenase level. When a response was achieved, a slow plasma exchange taper was initiated, usually involving every other day, three times weekly, or twice weekly schedules before cessation of treatment. This was at the discretion of the treating physician.

### Response and relapse criteria

A complete response to plasma exchange was defined as a platelet count greater than 100,000/μL for two consecutive evaluations, declining lactate dehydrogenase levels (if initially elevated), and no further neurological deficits or progression. A partial response was defined as stabilization of the platelet count below 100,000/μL with no further neurological deficits or progression. To allow comparisons with previous reports using 150,000 as the response cutoff, we analyzed our data using this level as well. Patients who had progressive thrombocytopenia, worsening neurological deficits, or clinical deterioration while undergoing plasma exchange therapy were deemed treatment failures. Relapse (after a documented response) was defined as the recurrence of any or all of the following: the initial signs and symptoms, MAHA (associated with an increase of LDH to > 500 U/L), thrombocytopenia (< 100,000/μL), and abrupt or slowly progressive deterioration in neurological status following cessation of plasma exchanges. Responding patients who failed to follow-up after the initial hospitalization for TTP-HUS were censored from relapse determination. Neurologic impairment included headaches, mental status changes (including confusion, obtundation, and coma), acute sensory or motor deficits, and seizures.

### Statistical analyses

Primary outcomes for this study included 30-day mortality and relapse, with specific interest in the associations between patient characteristics at the time of diagnosis of TTP or HUS and these outcomes. Logistic regression was the main model used to assess these associations. We began by investigating the univariate associations, followed by a multivariate model that included all patient characteristics variables. Odds ratios (OR) and 95% confidence intervals (CI) are reported. In addition, Kaplan-Meier curves were used to investigate long-term survival for patients with and without serious underlying disorders (i.e., cancer, solid organ transplantation, HIV infection, sepsis, hepatitis C, and *E. coli *O157:H7 infection), and a log-rank test was used to test for differences in the survival patterns. All analyses were performed using SAS, with a p-value < 0.05 considered statistically significant. Continuous data are reported as means ± SD, unless otherwise noted.

## Competing interests

The authors declare that they have no competing interests.

## Authors' contributions

ML data acquisition and analysis; first draft; editing and final draft approval.  DH data analysis; editing. PL concept and design; data acquisition; final draft approval.  TW concept and design; data analysis; final draft approval.
